# Increased protein phosphatase 5 expression in inflammation-induced left ventricular dysfunction in rats

**DOI:** 10.1186/s12872-022-02977-z

**Published:** 2022-12-09

**Authors:** Ashmeetha Manilall, Lebogang Mokotedi, Sulè Gunter, Regina Le Roux, Serena Fourie, Colleen A. Flanagan, Aletta M. E. Millen

**Affiliations:** Integrated Molecular Physiology Research Initiative, School of Physiology, Faculty of Health Sciences, University of the Witwatersrand, 7 York Road, Parktown, Johannesburg, 2193 South Africa

**Keywords:** Left ventricular diastolic dysfunction, Microvascular inflammation, Titin phosphorylation, Tumor necrosis factor-alpha inhibitor, Protein phosphatase 5

## Abstract

**Background:**

Titin phosphorylation contributes to left ventricular (LV) diastolic dysfunction. The independent effects of inflammation on the molecular pathways that regulate titin phosphorylation are unclear.

**Methods:**

We investigated the effects of collagen-induced inflammation and subsequent tumor necrosis factor-α (TNF-α) inhibition on mRNA expression of genes involved in regulating titin phosphorylation in 70 Sprague-Dawley rats. LV diastolic function was assessed with echocardiography. Circulating inflammatory markers were quantified by enzyme-linked immunosorbent assay and relative LV gene expression was assessed by Taqman® polymerase chain reaction. Differences in normally distributed variables between the groups were determined by two-way analysis of variance (ANOVA), followed by Tukey *post-hoc* tests. For non-normally distributed variables, group differences were determined by Kruskal–Wallis tests.

**Results:**

Collagen inoculation increased LV relative mRNA expression of vascular cell adhesion molecule 1 (*VCAM1*), pentraxin 3 (*PTX3*), and inducible nitric oxide synthase (*iNOS*) compared to controls, indicating local microvascular inflammation. Collagen inoculation decreased soluble guanylate cyclase alpha-2 (*sGCα2*) and soluble guanylate cyclase beta-2 (*sGCβ2*) expression, suggesting downregulation of nitric oxide-soluble guanylate cyclase-cyclic guanosine monophosphate (NO-sGC-cGMP) signaling. Inhibiting TNF-α prevented collagen-induced changes in *VCAM1*, *iNOS*, *sGCα2* and *sGCβ2* expression. Collagen inoculation increased protein phosphatase 5 (*PP5*) expression. Like LV diastolic dysfunction, increased *PP5* expression was not prevented by TNF-α inhibition.

**Conclusion:**

Inflammation-induced LV diastolic dysfunction may be mediated by a TNF-α-independent increase in *PP5* expression and dephosphorylation of the N2-Bus stretch element of titin, rather than by TNF-α-induced downregulation of NO-sGC-cGMP pathway-dependent titin phosphorylation. The steady rise in number of patients with inflammation-induced diastolic dysfunction, coupled with low success rates of current therapies warrants a better understanding of the systemic signals and molecular pathways responsible for decreased titin phosphorylation in development of LV diastolic dysfunction. The therapeutic potential of inhibiting PP5 upregulation in LV diastolic dysfunction requires investigation.

## Background

Heart failure with a preserved ejection fraction (HFpEF) accounts for approximately 50% of heart failure cases [1]. Chronic systemic inflammation associated with comorbid conditions such as obesity and diabetes mellitus has been implicated in the development of left ventricular (LV) diastolic dysfunction, the condition that frequently precedes HFpEF [2–5]. It has been proposed that inflammation contributes to LV diastolic dysfunction by disrupting phosphorylation of the giant spring protein, titin [6]. Decreased titin phosphorylation increases sarcomere tension contributing to deceased cardiomyocyte compliance and hence increases passive cardiomyocyte stiffness, impairing myocardial relaxation [6–9].

Chronic systemic inflammation increases circulating concentrations of pro-inflammatory proteins that are thought to disrupt myocardial signaling and cardiomyocyte function by upregulating cardiac microvascular endothelial cell expression of cell adhesion proteins [2, 3]. These adhesion molecules enhance trans-endothelial movement of leukocytes and stimulate production of reactive oxygen species (ROS) [10], which disrupt nitric oxide (NO) signaling [2]. NO released from endothelial cells enters cardiomyocytes, activating soluble guanylate cyclase (sGC) to produce cGMP, which activates protein kinase G (PKG) [11]. Activated PKG phosphorylates multiple sites within the N2-B spring element (N2-Bus) of titin [12, 13]. Phosphorylation of the N2-Bus by PKG and other kinases decreases titin-based passive tension within cardiomyocytes [14]. Thus, decreased NO availability has been proposed to result in hypophosphorylation of titin, which contributes to increased myocardial stiffness [3].

Titin function is also regulated by protein phosphatase enzymes, which dephosphorylate the N2-Bus, increasing sarcomere tension. Protein phosphatases PP1 and PP2A have been implicated in inflammation-induced dephosphorylation of titin in obese rats with HFpEF [2]. However, these phosphatases are stimulated by metabolic signals [15], hence their contribution to regulating titin phosphorylation independent of metabolic signals is uncertain.

Protein phosphatase (PP) 5 specifically dephosphorylates the titin N2-Bus region. Increased expression of PP5 increased dephosphorylation of the titin N2-Bus in vitro and in vivo and increased cardiomyocyte passive tension [16]. It was suggested that “pathological triggers” may activate PP5 in heart disease [16]. Although the proximal intracellular signaling molecules that activate PP5 have been reported [16, 17], it is unknown whether systemic inflammation affects PP5 activity or expression.

It has been suggested that inflammation associated with comorbid conditions such as hypertension, obesity and diabetes mellitus, causes LV diastolic dysfunction and HFpEF [3]. However, the effects of insulin, pressure and volume overload in these comorbidities confound understanding of the inflammation-induced effects on the protein kinase pathways that mediate phosphorylation of titin and the protein phosphatases that dephosphorylate titin [18]. Nevertheless, patients with systemic inflammatory conditions, such as rheumatoid arthritis, are at increased risk of developing LV diastolic dysfunction and HFpEF, independent of comorbid diseases [19, 20]. Although inflammation has been directly linked to the development of LV diastolic dysfunction in patients with rheumatoid arthritis [21], the effects of tumor necrosis factor alpha (TNF-α) inhibitors on LV diastolic dysfunction and HFpEF have been contradictory [22]. Therefore, this study used the collagen-induced arthritis (CIA) rat model to determine the independent effects of inflammation on expression of genes that regulate titin phosphorylation and development of LV diastolic dysfunction. We also used the TNF-α inhibitor, etanercept, to dissect the role of this proinflammatory cytokine in the pathways that regulate titin phosphorylation.

## Methods

### Animal treatment and confirmation of systemic inflammation and LV diastolic dysfunction

Experimental procedures were approved by the Animal Ethics Screening Committee of the University of the Witwatersrand (AESC approval: 2017/03/21 C and 2019/02/10 C). Procedures were performed in compliance with the Guide for the Care and Use of Laboratory Animals and reported in accordance with the ARRIVE guidelines. Three-month-old Sprague-Dawley rats were obtained from the Wits Research Animal Facility. Rats were randomly assigned to the control or experimental group using an online random number generator. The control group (n = 26, males n = 14, females n = 12) received a subcutaneous injection of saline (0.2 ml). A modified Chondrex collagen inoculation protocol was employed to induce systemic inflammation in the experimental group, as previously described [23, 24]. Briefly, the experimental group was injected subcutaneously at the base of the tail with Bovine type II collagen (2 mg/ml in 0.05 M acetic acid) emulsified in incomplete Freund’s adjuvant (1:1 volume, 0.2 ml). Booster injections (0.1ml) were administered at seven and 21 days. Collagen-inoculated rats with confirmed arthritis were further randomized into the CIA group (n = 28, males n = 13, females n = 15) that did not receive further treatment and the CIA + TNF-α inhibitor group (CIA + anti-TNF-α; n = 16, males n = 8, females n = 8) that were treated with etanercept, a TNF-α inhibitor (Enbrel, 10 mg/kg^−1^, Amgen Inc., Thousand Oaks, CA) every three days for 6 weeks. The lower number of rats in the CIA + anti-TNF-α group was due to the expense of the etanercept. The non-invasive tail-cuff technique was used to measure blood pressure biweekly (Biopac Systems, Santa Barbara, CA, USA). The rats used in the current study are part of a larger study of the effects of collagen-induced systemic inflammation and, in accordance with ethics committee recommendations to minimize numbers of animals used, echocardiography data for a subset (n = 64) of the rats used in the current study have been previously reported [24].

Rats were anaesthetized by intramuscular injections of ketamine (100 mg kg^−1^) and xylazine (5 mg kg^−1^). LV diastolic function was determined by echocardiography (Acuson SC 2000, Siemens Medical Solutions, USA, Inc.) by an experienced observer who was blinded to the treatment groups, as previously described [24, 25]. Briefly, LV diastolic function was assessed using pulsed Doppler, tissue Doppler imaging (TDI) and left atrial volume. Pulsed Doppler was used to record transmitral flow patterns at the mitral valve leaflet tips in the apical four chamber view. The early (E) and late atrial (A) diastolic waves were measured from the mitral valve inflow velocity curve. LV stiffness was expressed as the lateral wall e′/a′ ratio. LV relaxation and LV filling pressure were indexed by lateral wall e′ and E/e′, respectively [24, 25]. Following echocardiography, rats were sacrificed with an overdose of anaesthesia (ketamine 200 mg kg^−1^ and xylazine 10 mg kg^−1^) followed by thoracotomy and the left ventricle of the hearts was dissected and stored (− 80 °C) in RNA*later®* (Ambion, Sigma Aldrich, Germany). Blood was sampled and allowed to clot for 2 h at room temperature. Blood was centrifuged and serum was collected and stored at − 80 °C. Sandwich enzyme-linked immunosorbent assays (ELISA, Elabscience Biotechnology, USA) were used for duplicate measurements of serum CRP, TNF-α, and IL-6 concentrations [24, 26].

### Comparative gene expression PCR

Total RNA was extracted from rat LV tissues using an illustra™ Mini Spin RNA extraction kit (GE Healthcare, Buckinghamshire) and reverse-transcribed into cDNA using SuperScript™ IV VILO™ cDNA synthesis master mix (Thermo Fisher Scientific, Life Technologies, Carlsbad, USA). Comparative gene expression RT-PCR was performed on a StepOne Plus thermocycler (Thermo Fisher Scientific, Life Technologies, Carlsbad, USA) using standard cycling conditions.

Comparative gene expression of the following genes was assessed: *VCAM1* which codes for VCAM1 protein (Taqman assay ID: Rn00563627_m1), *PTX3* which codes for PTX3 protein (Taqman assay ID: Rn01769850_m1), *iNOS* which codes for inducible nitric oxide synthase (iNOS) protein (Taqman assay ID: Rn00561646_m1), *PP1γ*, which codes for PP1 protein phosphatase (Taqman assay ID: Rn04339209_m1), *PP5*, which codes for PP5 protein phosphatase (Taqman assay ID: Rn00669044_g1), *sGCα2* (Taqman assay ID: Rn00675050_m1) and *sGCβ2*, (Taqman assay ID: Rn01636981_m1) which code for the α2 and β2 subunits of the sGC protein respectively. Duplex reactions (10 µl) were performed using cDNA (~ 2 µg), pre-designed target TaqMan probe mixes (0.5 µl, TaqMan gene expression assay) and TaqMan advanced PCR master mix (5 µl) with hypoxanthine phosphoribosyltransferase 1 (*HPRT1*) (0.25 µl, TaqMan assay ID: Rn01527838_g1) in duplicated plates. The delta-delta Ct method was used to calculate the fold-change in relative expression using the formula, 2^−∆∆Ct^ [27]. When results for individual rats were outside the detection range of the PCR assay in duplicate plates, these data points were excluded from the analysis.

### Data analysis

SAS software, version 9.4 (SAS Institute Inc., USA) was used for statistical analyses. Non-normally distributed variables are expressed as median (interquartile range, IQR). Normally distributed variables are expressed as means ± SEM. Changes in body mass over time and differences between groups were determined by repeated measures analysis of variance (ANOVA), including sex as a potential confounder in the model. For normally distributed variables, group differences at termination were determined by two-way ANOVA using the generalized linear model (GLM procedure in SAS), with group and sex as main effects, followed by Tukey *post-hoc* tests. In addition, to account for the potential impact of differences in body mass between the groups, body mass was included as an additional confounder in a separate model. The GLM procedure allows unbalanced data and the inclusion of continuous variables as covariates. For non-normally distributed data, group differences were determined by Kruskal–Wallis tests. As there were no differences in gene expression data between males and females, results were combined. Results were considered statistically significant if p ≤ 0.05.

## Results

### Body mass, blood pressure, markers of inflammation and diastolic function measures

The characterization of the animal model has been reported in a partially overlapping group of rats [24]. Briefly, for the animals included in this study, body mass was not different at baseline between the groups, and the body mass increased over the duration of the study in all groups compared to baseline (all p < 0.05). Compared to controls, body mass at termination was lower in the CIA + anti-TNF-α (p = 0.005) group, as well as in the CIA group, but it did not reach statistical significance (p = 0.07) (Table 1). The lower terminal body mass in the CIA + anti-TNF-α group suggests that increased circulating TNF-α does not contribute to lower mass gain in collagen-inoculated groups compared to controls. There were no differences in blood pressure between the groups (Table 1). Circulating TNF-α (CIA; p < 0.0001; CIA + anti-TNF-α; p < 0.0001), IL-6 (CIA; p < 0.0001; CIA + anti-TNF-α; p < 0.001) and CRP (CIA; p < 0.0001; CIA + anti-TNF-α; p < 0.0001) concentrations were higher in both groups of rats inoculated with collagen compared to control rats (Table 1), confirming induction of systemic inflammation. Circulating CRP concentrations were lower in the CIA + anti-TNF-α group compared to the CIA group (p = 0.05). LV diastolic function was significantly impaired in the CIA and CIA + anti-TNF-α groups (Table 1), as previously reported [24, 26]. Briefly, lateral wall e′, e′/a′ and E/e′ were significantly impaired in both groups of rats inoculated with collagen compared to the control group (Table 1).

**Table 1:** Body mass, blood pressure, circulating inflammatory marker concentrations and echocardiographic measures of diastolic function

	Control	CIA	CIA + anti-TNF-α	
n	26	28	16	
Female n (%)	12 (46)	15 (54)	8 (50)	
Body mass at baseline (g)	393.7 ± 8.8	380.6 ± 8.0	382.5 ± 11.3	
Body mass at termination (g)	451.7 ± 8.8	420.5 ± 8.0	400.0 ± 11.3*	
SBP (mm Hg) DBP (mm Hg)	128 ± 2 87 ± 2	130 ± 2 88 ± 1	132 ± 2 86 ± 2	
*Circulating inflammatory marker concentrations*				
TNF-α (pg/ml)	91.2 ± 5.0	159.9 ± 7.0* (1.75 fold)^a^	146.2 ± 9.6* (1.59 fold)	
IL-6 (pg/ml)	16.0 ± 1.8	31.1 ± 2.0* (1.94 fold)	27.9 ± 2.6* (1.75 fold)	
CRP (ng/ml)	0.09 ± 0.03	0.58 ± 0.04* (6.4 fold)	0.43 ± 0.05*^#^ (4.8 fold)	
*Left ventricular diastolic function measures*				
e′ (cm/s)	4.52 ± 0.11	3.60 ± 0.09*	3.50 ± 0.11*	
e′/a′	1.44 ± 0.06	1.27 ± 0.07*	1.04 ± 0.04*	
E/e′	25.12 ± 1.25	31.61 ± 1.34*	32.52 ± 1.55*	

Data are expressed as means ± SEM. CIA, collagen-induced arthritis, anti-TNF-α, collagen-inoculated and treated with tumor necrosis factor-alpha inhibitor

*SBP* systolic blood pressure, *DBP* diastolic blood pressure, *TNF-α* tumor necrosis factor alpha, *IL-6* interleukin 6, *CRP* C-reactive protein

*p < 0.05 versus control; ^#^p < 0.05 versus CIA

^a^Fold-change values for circulating inflammatory marker concentrations compared to control are shown in parenthesis

### Expression of genes involved in microvascular inflammation and immune cell activation

**Fig. 1:**
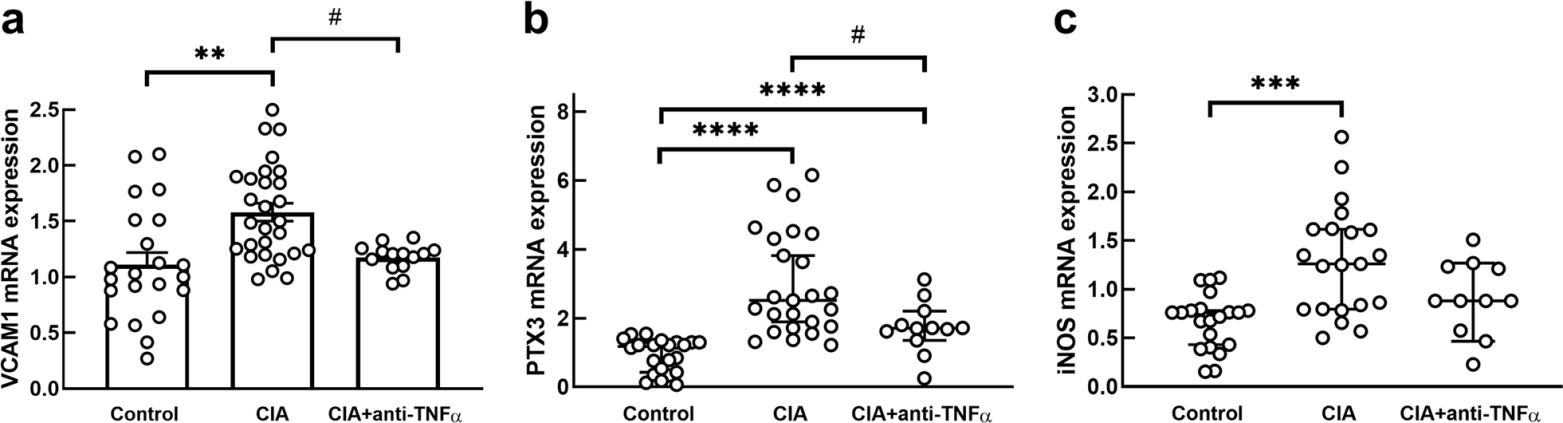
LV expression of markers of microvascular inflammation and immune cell activation. Relative mRNA expression of *VCAM1* (**a**), *PTX3* (**b**) and *iNOS* (**c**) in left ventricular tissues of control, CIA and CIA + anti-TNFα. Results are expressed in arbitrary units normalized for the expression of the endogenous reference gene, *HPRT1*, using the ∆∆Ct method. Data are presented as mean ± SEM for *VCAM1* and median and interquartile range for *PTX3* and *iNOS*; **p < 0.01 versus control, ***p < 0.001 versus control, ****p < 0.0001 versus control, ^#^p < 0.05 versus CIA

The mean ± SEM relative LV mRNA expression of *VCAM1*, a marker of endothelial activation, was significantly increased in the CIA group (1.55 ± 0.08; p = 0.007), but not in the CIA + anti-TNF-α group (1.18 ± 0.11; p = 0.98) compared to the control group (1.16 ± 0.09, Fig. 1a). The relative mRNA expression of *VCAM1* was significantly lower in the CIA + anti-TNF-α group compared to the CIA group (p = 0.03, Fig. 1a). The relative LV mRNA expression of *PTX3*, a marker of local inflammation, [median (IQR)] was significantly higher in the CIA (2.40 (1.74–4.06); p < 0.0001) and the CIA + anti-TNF-α (1.71 (1.48–2.00); p = 0.001) groups compared to the control group (1.18 (0.43–1.3, Fig. 1b). The relative mRNA expression of *PTX3* in the CIA + anti-TNF-α group was significantly lower than in the CIA group (p = 0.05, Fig. 1b). The relative mRNA expression of *iNOS*, a marker of infiltrating leukocytes, was significantly increased in the CIA group (median (IQR); 1.26 (0.80–1.62); p = 0.0003) compared to the control group (0.76 (0.43–0.78), but not in the CIA + anti-TNF-α group (0.88 (0.58–1.23); p = 0.10) (Fig. 1c). When including body mass as a potential confounder in the model, the results were materially unaltered.

### Inflammation effects on NO signaling pathway genes

**Fig. 2:**
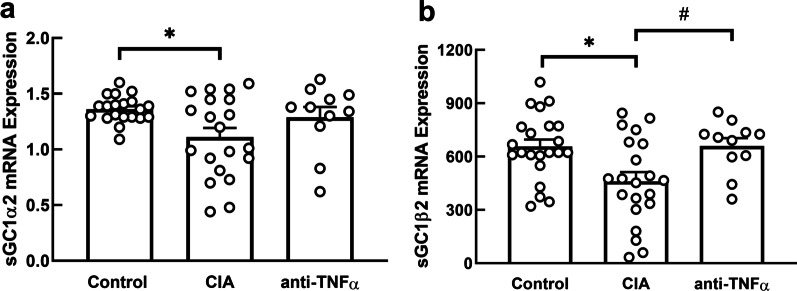
LV expression of sGC subunit genes. Relative mRNA expression of *sGCα2* (**a**) and *sGCβ2* (**b**) in left ventricular tissues of control, CIA, and CIA + anti-TNF-α. Results are expressed in arbitrary units normalized for the expression of the endogenous reference gene, *HPRT1*, using the ∆∆Ct method. Data expressed as mean ± SEM; *p < 0.05 versus control; ^#^p < 0.05 versus CIA

The mean ± SEM relative mRNA expression of *sGCα2* (Fig. 2a) and *sGCβ2* (Fig. 2b), the isoforms that code for the sGC enzyme that initiates NO-stimulated cGMP signaling, were significantly decreased in the CIA group (*sGCα2*: 1.10 ± 0.06; *sGCβ2*: 453.3 ± 44.8) compared to control group (*sGCα2*: 1.37 ± 0.06, p = 0.02, *sGCβ2*: 641.8 ± 43.3, p = 0.01). The relative mRNA expression of *sGCα2* and *sGCβ2* in the CIA + anti-TNF-α rats (1.32 ± 0.09; p = 0.13, 674.9 ± 62.4, p = 0.90, respectively) were not different from controls (Fig. 2). The relative mRNA expression of *sGCβ2* was significantly increased in the CIA + anti-TNF-α group compared to the CIA group (p = 0.02, Fig. 2b). When including body mass as a potential confounder in the model, the results were materially unaltered.

### Inflammation effects on protein phosphatases

**Fig. 3:**
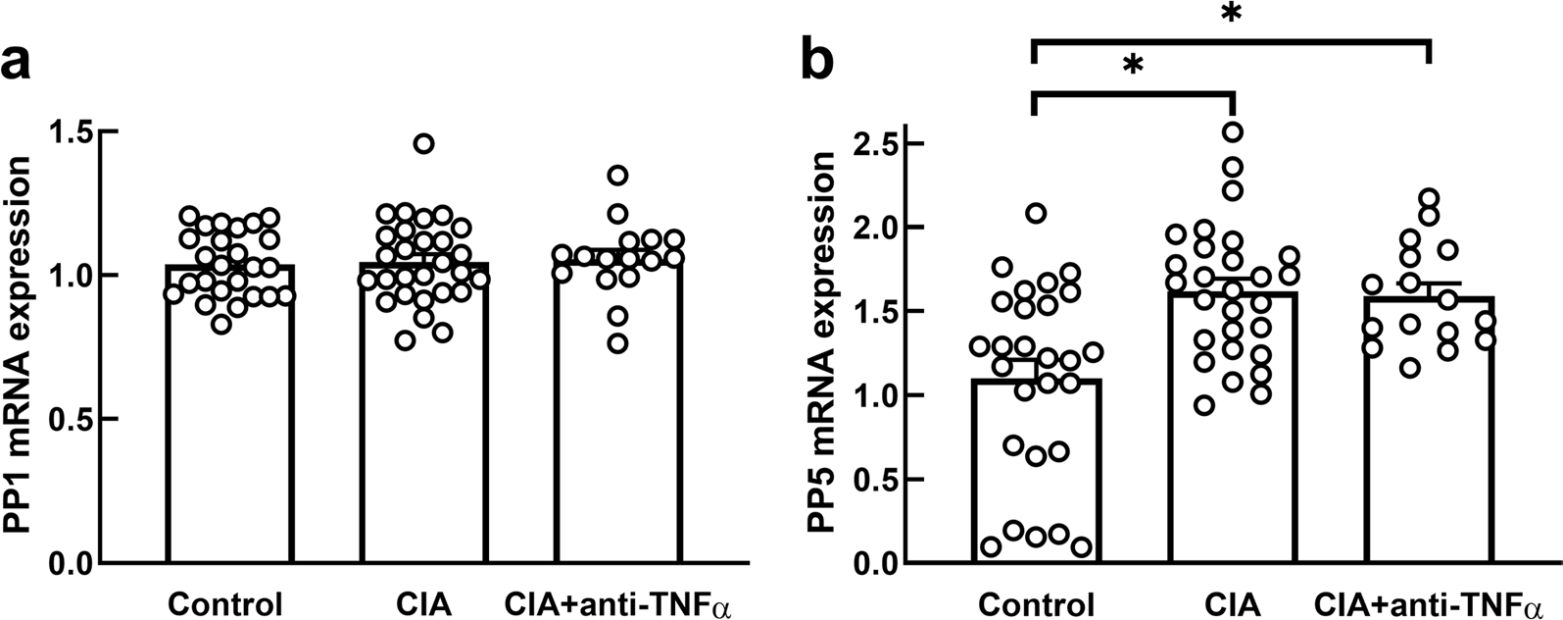
LV expression of protein phosphatases. Relative mRNA expression of *PP1γ* (**a**) and *PP5* (**b**) in left ventricular tissues of control, CIA and CIA + anti-TNF-α. Results are expressed in arbitrary units normalized for the expression of the endogenous reference gene, *HPRT1*, using the ∆∆Ct method. Data expressed as mean ± SEM; * p < 0.05 versus control group

The mean ± SEM relative mRNA expression of *PP1γ*, the protein phosphatase previously implicated in titin phosphorylation, was not different between the three groups (p > 0.05, Fig. 3a). The relative mRNA expression of the protein phosphatase *PP5* was significantly higher in both groups of rats inoculated with collagen (CIA, 1.61 ± 0.09, p = 0.008; CIA + anti-TNF-α, 1.59 ± 0.11, p = 0.04), compared to the control group (1.24 ± 0.09, Fig. 3b) and was not different between the CIA and CIA + anti-TNF-α groups (p > 0.05). When including body mass as a potential confounder in the model, the results were materially unaltered.

## Discussion

This study investigated the effects of chronic systemic inflammation, in the absence of metabolic confounders, on expression of genes involved in LV microvascular endothelial activation, NO-sGC-cGMP signaling and titin dephosphorylation, implicated in LV diastolic dysfunction. We show that collagen inoculation increased LV expression of *VCAM1*, suggesting endothelial activation. Furthermore, we show increased LV expression of *PTX3* and *iNOS* indicating increased local tissue inflammation and leukocyte invasion. Supporting the role of decreased NO-dependent signaling in development of LV diastolic dysfunction, collagen inoculation decreased the expression *sGCα2* and *sGCβ2*, which code for isoforms of the sGC enzyme that initiates NO-stimulated cGMP signaling. The increased expression of *VCAM1*, *PTX3* and *iNOS*, together with decreased expression of sGC were prevented by treatment with a TNF-α inhibitor. Taken together, these results show that increased circulating TNF-α stimulates vascular inflammation, increases LV tissue inflammation and disrupts NO-sGC-cGMP signaling, which may modulate cardiomyocyte elasticity, likely by phosphorylating the titin N2-Bus. However, the collagen-induced LV diastolic dysfunction is not reversed by blocking circulating TNF-α [24], suggesting that TNF-α and hence, decreased NO-sGC-cGMP signaling is likely not the primary mechanism of inflammation-induced LV diastolic dysfunction. Collagen inoculation had no effect on expression of *PP1γ*, but it significantly increased expression of *PP5*, a major catalyst of titin dephosphorylation [16]. Like the inflammation-induced LV diastolic dysfunction, the inflammation-induced up-regulation of *PP5* expression was not reversed by inhibiting circulating TNF-α. These results show that increased expression of *PP5* may increase cardiomyocyte stiffness by decreasing the net phosphorylation level of the N2-Bus stretch element of titin, which may partially mediate chronic systemic inflammation-induced LV diastolic dysfunction.

A recent paradigm suggested that in response to increased circulating inflammatory cytokines, microvascular inflammation drives altered myocardial signaling that ultimately results in hypophosphorylation of the titin N2-Bus and consequently decreased myocyte compliance [3]. The paradigm was supported by an obese diabetic rat model of metabolic risk-associated systemic inflammation, which showed evidence of microvascular inflammation and disruption of NO signaling [2]. Our model of collagen-induced systemic inflammation, which is independent of metabolic confounders, showed upregulated LV mRNA expression of *VCAM1*. VCAM1 is a glycoprotein that is expressed predominantly on the surfaces of endothelial cells and is activated by pro-inflammatory cytokines [10, 28]. Microvascular inflammation resulting from increased expression of cell adhesion molecules, including VCAM1, promotes leukocyte transmigration [29, 30] and enhances ROS production, which are characteristic of an oxidative stress environment [31–33]. Supporting this, we previously showed that collagen inoculation increased *CD68* expression in LV tissues, showing infiltration of leukocytes [26]. In the present study, *PTX3* and *iNOS* expression was increased in CIA rats, further indicating leukocyte infiltration in the heart tissues and hence local inflammation. PTX3 is an acute-phase protein that is structurally similar to CRP [34], and is produced by inflammatory cells, such as monocytes and neutrophils, but it is also produced locally by vascular endothelial cells at the sites of inflammation [35]. Leukocytes that express *iNOS* are known for their production of ROS, which disrupt NO bioavailability by facilitating dissociation of endothelial NOS dimers [36, 37] and by reacting with NO to produce peroxynitrite [38]. Thus, microvascular inflammation causes the transmigration of immune cells that increase local production of ROS, which interfere with NO–sGC signaling by reducing NO bioavailability [39].

NO diffuses into cardiomyocytes, where it activates sGC to produce cGMP. cGMP, in turn, activates PKG, one of the kinases that decrease sarcomere tension by phosphorylating the titin N2-Bus region. Whereas decreased local NO concentrations would result in decreased NO-sGC-cGMP signaling, it has been reported that ROS also decrease the responsiveness of the NO-sGC-cGMP system to exogenous NO donors, suggesting that decreased activity or expression of sGC may further decrease cGMP signaling [7]. ROS have been shown to disrupt cGMP production by oxidizing the heme required for NO to bind to sGC and by decreasing protein and mRNA expression of sGC subunits [7, 11, 40]. We have shown that systemic inflammation decreased mRNA expression of both the *sGCα2* and *sGCβ2* genes. This suggests that the decreased *sGCα2* and *sGCβ2* expression induced by collagen inoculation is likely mediated by increased ROS production and that the decreased expression will result in decreased NO-sGC-cGMP signaling [41].

In the current study, blocking circulating TNF-α prevented the inflammation-induced upregulation of *VCAM1, PTX3* and *iNOS* and therefore arguably inhibited pathological ROS production. Furthermore, blocking TNF-α also prevented the inflammation-induced downregulation of mRNA expression of sGC genes. Taken together, our results suggest that increased circulating TNF-α increases LV microvascular endothelial activation, which increases ROS production by endothelial cells and via immune cell transmigration. The resultant increased ROS decrease sGC expression and likely also decrease its activity [7, 40]. This will result in decreased cGMP signaling and hence decreased PKG phosphorylation of the titin N2-Bus region. However, despite the impact of TNF-α blockade on microvascular inflammation and NO-dependent cGMP signaling, we have shown that LV diastolic dysfunction induced by collagen inoculation was not reversed by blocking circulating TNF-α [24]. This indicates that TNF-α and hence, decreased cGMP-stimulated phosphorylation of titin, may not be the primary mechanisms of LV diastolic dysfunction induced by systemic inflammation. In this regard, it is important to consider that kinases other than NO-dependent PKG phosphorylate the N2-Bus region of titin [42] and that the net phosphorylation state of titin is also regulated by the phosphatases that dephosphorylate the residues phosphorylated by the kinases, increasing titin stiffness [43]. Like protein kinases, protein phosphatases are also regulated by systemic and intracellular signaling pathways [44].

Phosphorylation and dephosphorylation of titin are part of normal cardiomyocyte contraction. Increased expression of protein phosphatases causes decreased net phosphorylation of cardiac proteins [45]. It has been suggested that increased expression of protein phosphatases PP1 and PP2A may contribute to the hypophosphorylation of titin, characteristic of LV diastolic dysfunction [2]. In the present study, we showed that the relative mRNA expression of *PP1γ*, which codes for a major eukaryotic protein serine/threonine phosphatase that regulates a variety of cellular functions [15, 45, 46], was not altered in response to systemic inflammation or TNF-α inhibitor treatment. This is in contrast to a previous study that reported increased PP1 protein expression in rats that developed LV diastolic dysfunction in a model of obesity-associated inflammation [2]. Considering the well-known impact of metabolic signals on the regulation of PP1 expression [15, 47], the increased PP1 expression in the prior study may be explained by the metabolic dysfunction likely to result from obesity [2]. Therefore, the unchanged expression of *PP1γ* in our model of systemic inflammation independent of metabolic dysfunction suggests that PP1 expression may not be directly regulated by chronic systemic inflammation.

The N2-Bus region of titin was recently found to also be dephosphorylated by PP5 [16]. PP5 is ubiquitously expressed in eukaryotic cells and its activity is enhanced by ROS [16, 44]. PP5 expression was shown to be increased in tissue from end-stage failing human hearts and from dogs with LV diastolic dysfunction [16]. However, the stimulus for the increased PP5 expression in LV diastolic dysfunction and heart failure was not explored. In the current study we showed that systemic inflammation, independent of metabolic dysfunction, increased relative mRNA expression of *PP5* in collagen-inoculated rats with LV diastolic dysfunction. Our results indicate that systemic inflammation-induced LV diastolic dysfunction may, at least in part, be mediated by increased PP5 expression and dephosphorylation of titin. However, other phosphoprotein phosphatases such as PP2C [48] and PP2A [2], that were not measured, may also be involved in titin phosphorylation. Furthermore, molecular pathways independent of titin compliance including myocardial fibrosis and calcium handling may contribute to LV diastolic dysfunction in this study [24, 42].

Importantly, in contrast to the NO-sGC-cGMP pathway, the inflammation-induced upregulation of *PP5* expression was not reversed by blocking circulating TNF-α. This suggests that the local *PP5* expression may be regulated by a circulating inflammatory signal other than TNF-α. Although PP5 expression is known to be regulated by several intracellular signals [16, 17], the extracellular signals that stimulate these pathways to PP5 expression and activity require further investigation. Since both the upregulation of *PP5* expression and the accompanying LV diastolic dysfunction are not mediated by TNF-α, our results suggest that LV diastolic dysfunction may, at least in part, result from increased expression of PP5, which increases cardiomyocyte stiffness by decreasing the net phosphorylation level of the N2-Bus stretch element of titin, whereas disruption of the NO-sGC-cGMP pathway is likely less important.

This study has some limitations. The inclusion of both male and female rats may have reduced statistical power. The use of mRNA expression may not reflect protein expression and function, hence direct measures of NO, ROS and titin phosphorylation may have strengthened the interpretations of this study, and should be included in future studies. Although etanercept has been successfully used in previous animal models [33], it is a human fusion protein, which, itself, may elicit an immunogenic response in rats.

## Conclusion

In summary, our results show that systemic inflammation induced by collagen inoculation, contributes to microvascular endothelial inflammation and reduced *sGC* expression, possibly disrupting the NO-sGC-cGMP signaling pathway that increases titin phosphorylation. Our findings suggest that pathological downregulation of NO-sGC-cGMP signaling may not be the primary signal for hypophosphorylation of the titin N2-Bus in HFpEF [3, 26], since blocking circulating TNF-α prevented the inflammation-induced effects on NO-sGC-cGMP signaling, but had no effect on LV diastolic dysfunction. Like LV diastolic dysfunction, the inflammation-induced increase in *PP5* expression was not prevented by inhibiting circulating TNF-α. Therefore, our results suggest that the activity of PP5 is likely a mediator of inflammation-induced LV stiffness and that inflammatory signals other than TNF-α regulate *PP5* expression. Future studies should explore the systemic inflammatory signals responsible for increased *PP5* expression. Furthermore, although etanercept treatment is useful for decreasing inflammatory symptoms in patients with rheumatoid arthritis, it may not modulate cardiac expression of *PP5* and its effects on the development of HFpEF. The therapeutic potential of inhibiting PP5 in the management of LV diastolic dysfunction warrants investigation.

## Data Availability

The datasets generated during and/or analyzed during the current study are available from the corresponding author on reasonable request.

## Abbreviations

A: Mitral inflow velocity during late diastole
a′: Mitral annular tissue lengthening during late diastole
ANOVA: Analysis of variance
CD68: Cluster of differentiation 68
cGMP: Cyclic guanosine monophosphate
CIA: Collagen-induced inflammation
CRP: C-reactive protein
DBP: Diastolic blood pressure
E: Mitral inflow velocity during early diastole
e′: Mitral annular tissue lengthening during early diastole
GMP: Guanosine monophosphate
HFpEF: Heart failure with a preserved ejection fraction
HFrEF: Heart failure with a reduced ejection fraction
HPRT1: Hypoxanthine phosphoribosyltransferase 1
IL-6: Interleukin-6
iNOS: Inducible nitric oxide synthase
IQR: Interquartile range
LV: Left ventricular
NO: Nitric oxide
PCR: Polymerase chain reaction
PKG: Protein kinase G
PP: Protein phosphatase
PTX3: Pentraxin 3
ROS: Reactive oxygen species
RT-PCR: Real time polymerase chain reaction
SBP: Systolic blood pressure
SEM: Standard error of the mean
sGC: Soluble guanylate cyclase
TNF-α: Tumor necrosis factor alpha
VCAM1: Vascular cell adhesion molecule 1
